# Exploring the immune escape mechanisms in gastric cancer patients based on the deep AI algorithms and single‐cell sequencing analysis

**DOI:** 10.1111/jcmm.18379

**Published:** 2024-05-16

**Authors:** Wenli Chen, Xiaohui Liu, Houhong Wang, Jingyou Dai, Changquan Li, Yanyan Hao, Dandan Jiang

**Affiliations:** ^1^ Department of General Surgery The Affiliated Bozhou Hospital of Anhui Medical University Bozhou Anhui China; ^2^ Department of Nursing, Xiangya Hospital Central South University Changsha China; ^3^ Department of Pediatric Surgery The Affiliated Bozhou Hospital of Anhui Medical University Bozhou Anhui China; ^4^ Department of Articular Surgery The Affiliated Bozhou Hospital of Anhui Medical University Bozhou Anhui China; ^5^ The Second Affiliated Hospital, Department of Emergency, Hengyang Medical School University of South China Hengyang China

**Keywords:** AI algorithms, gastric adenocarcinoma, immune escape, macrophages, single‐cell analysis

## Abstract

Gastric cancer is a prevalent and deadly malignancy, and the response to immunotherapy varies among patients. This study aimed to develop a prognostic model for gastric cancer patients and investigate immune escape mechanisms using deep machine learning and single‐cell sequencing analysis. Data from public databases were analysed, and a prediction model was constructed using 101 algorithms. The high‐AIDPS group, characterized by increased AIDPS expression, exhibited worse survival, genomic variations and immune cell infiltration. These patients also showed immunotherapy tolerance. Treatment strategies targeting the high‐AIDPS group identified three potential drugs. Additionally, distinct cluster groups and upregulated AIDPS‐associated genes were observed in gastric adenocarcinoma cell lines. Inhibition of GHRL expression suppressed cancer cell activity, inhibited M2 polarization in macrophages and reduced invasiveness. Overall, AIDPS plays a critical role in gastric cancer prognosis, genomic variations, immune cell infiltration and immunotherapy response, and targeting GHRL expression holds promise for personalized treatment. These findings contribute to improved clinical management in gastric cancer.

## 1 INTRODUCTION

Gastric cancer is one of the most common and highly malignant tumours worldwide. According to statistics from the World Health Organization (WHO), gastric cancer is the third leading cause of cancer‐related death globally, causing approximately 720,000 deaths annually.^1^ Despite significant progress in the epidemiological research and clinical management of gastric cancer, many challenges still remain. Epidemiological data reveal regional variations in the incidence of gastric cancer, with particularly high rates in some Asian countries such as China, Japan and Korea.^2^ Furthermore, risk factors such as Helicobacter pylori infection, high salt diet, smoking and genetic factors are believed to play important roles in the development of gastric cancer.^3^, ^4^ Early diagnosis of gastric cancer is challenging, with many patients being diagnosed at advanced stages of the disease, leading to a lower success rate in treatment.^5^ Furthermore, traditional approaches encompassing radiotherapy, chemotherapy and surgical interventions exhibit noticeable limitations, particularly regarding treatment outcomes in advanced‐stage patients, while engendering drug resistance.^6^

Immunotherapy, as an emerging therapeutic strategy, has shown tremendous potential in the treatment of various malignancies in recent years.^7^ However, the application of immunotherapy in gastric cancer faces numerous challenges. Gastric cancer patients exhibit significant heterogeneity in their responsiveness and tolerability to immunotherapy, leading to considerable differences in individual treatment outcomes.^8^ Therefore, it is of great significance to gain in‐depth understanding of the immune molecular characteristics and immune evasion mechanisms in gastric cancer patients in order to develop more personalized treatment strategies.

In recent years, the combination of deep AI algorithms and single‐cell sequencing analysis has become an important tool for studying the immune features of gastric cancer patients.^9^, ^10^, ^11^, ^12^ Deep AI algorithms enable efficient processing and analysis of complex data, thereby uncovering immunologically relevant features with potential biological significance.^13^, ^14^ On the contrary, single‐cell sequencing provides high‐resolution information on cell types and states, revealing the distribution and function of immune cells in the gastric cancer microenvironment.^15^, ^16^ By combining these two methods, we can gain a more comprehensive understanding of the immune system status in gastric cancer patients and explore the molecular mechanisms underlying immune evasion.

In this study, we aim to comprehensively investigate the immune molecular characteristics and immune evasion‐related molecular mechanisms in gastric cancer patients through the integration of deep AI algorithms, single‐cell sequencing analysis and in vitro cellular experiments. Through a thorough analysis of immune cell subtypes and states in patients with gastric cancer, we aspire to offer more precise and tailored strategies for immune therapy in gastric cancer. This endeavour ultimately contributes to establish a solid foundation for subsequent research and clinical applications.

## 2 MATERIALS AND METHODS

### 2.1 Data acquisition and preprocessing for gastric adenocarcinoma

We collected the data set from public databases such as The Cancer Genome Atlas (TCGA) and Gene Expression Omnibus (GEO) following the steps below: (1) Collection of data sets with at least 40 samples and survival information; (2) Ensuring the data sets contained annotations for at least 15,000 genes; (3) Selection of patients who underwent preoperative treatment for primary tumours. Ultimately, we included a total of 1308 samples from four cohorts, including TCGA‐STAD (*n* = 375), GSE15459^17^ (*n* = 200), GSE62254^18^ (*n* = 300) and GSE84437^19^ (*n* = 433). The FPKM data for TCGA‐STAD was downloaded from the UCSC Xena database (https://xenabrowser.net/datapages/) and further transformed into log_2_ (TPM + 1) format. The exp‐Array data from GEO was standardized through their portal.

### 2.2 Univariate Cox regression analysis

Based on cross‐gene analysis, we performed univariate Cox regression analysis on the training cohort and three testing cohorts. Following the criteria below, we selected a set of genes with consistent prognostic effects as the target for further investigation: genes with a *p*‐value less than 0.05 and hazard ratio (HR) values greater than or less than 1.

### 2.3 AI‐predicted prognostic biomarkers

To create a consensus AIDPS (Artificial Intelligence‐based Prognostic Signature) that is highly accurate and stable, we combined 10 machine learning algorithms and 101 different algorithm combinations. These integrative algorithms included random survival forest (RSF), elastic network (Enet), Lasso, Ridge, stepwise Cox, CoxBoost, partial least squares regression for Cox (plsRcox), supervised principal components (SuperPC), generalized boosted regression modelling (GBM) and survival support vector machine (survival‐SVM). The procedure for generating the signature was as follows: (a) Prognostic genes were selected for further study based on the following criteria: a *p*‐value of less than 0.05 and hazard ratios (HRs) consistently greater than 1 or less than 1 in at least two out of four cohorts. (b) The prognostic genes were then used to build prediction models using the 101 algorithm combinations and the leave‐one‐out cross‐validation (LOOCV) framework in the TCGA‐STAD cohort. (c) All models were tested and evaluated using three external validation data sets (GSE15459, GSE62254 and GSE84437). (d) For each model, the Harrell's concordance index (C‐index) was calculated across all validation data sets, and the model with the highest average C‐index was deemed as the optimal model.

### 2.4 Identification of prognostic value of AIDPS

Utilizing the median, we stratified patients in the training cohort, the three testing cohorts and the meta cohort (which had batch effects and duplicates eliminated) into high and low AIDPS groups. The prognostic significance of AIDPS was assessed through Kaplan–Meier curves and multivariate Cox regression analysis. Furthermore, calibration curves and receiver operating characteristic (ROC) curves were generated to evaluate the predictive accuracy of AIDPS.

### 2.5 Evaluation of clinical significance and enrichment analysis of AIDPS

We compared key clinical characteristics, such as age, gender, TNM staging and grading, between the high AIDPS and low AIDPS groups. Using the Gene Set Enrichment Analysis (GSEA) method, we identified specific functional pathways in the high AIDPS and low AIDPS groups. After performing differential analysis using the DESeq2 package, we ranked all genes in descending order based on log_2_FoldChange (log_2_FC). Next, we utilized the clusterProfiler package to determine the enriched pathways of GO and KEGG and selected the top five pathways with higher normalized enrichment scores (NES) for visualization.

### 2.6 Genomic landscape alterations

To delve into the genomic variations in the high AIDPS group and low AIDPS group, a comprehensive analysis of mutations and copy number alterations (CNAs) was conducted using TCGA‐STAD data. Firstly, the raw mutation files were obtained, and tumour mutation burden (TMB) was calculated for each sample. The top 15 genes were visualized using the maftools package. Subsequently, the GISTIC 2.0 module in the Firebrowse tool was employed to identify and locate recurrent genomic amplifications and deletions. Finally, we selected broad regions with CNA frequency >20% on chromosomes 8q24.21, 9p21.3 and 18q21.2 to present.

### 2.7 Immune molecular expression and tumour microenvironment evaluation

The ssGSEA method was utilized to comprehensively estimate the immune and stromal component infiltration abundance in the high AIDPS group and low AIDPS group. Additionally, we examined the expression levels of 11 previously studied immune checkpoint molecules, including the B7‐CD28 family and TNF superfamily, in both groups. The immune inhibitory checkpoint tool was used to predict the responsiveness to immune responses in the high AIDPS group and low AIDPS group, with lower scores indicating better immunotherapy effects. Moreover, we calculated the gene expression similarity between patients in the high AIDPS group and low AIDPS group and those with response or no response to immune checkpoint inhibitors (ICIs) based on the results of Submap analysis to infer the efficacy of immune therapy.

### 2.8 Development and validation of potential therapeutic drugs

Potential therapeutic drugs were developed targeting the high AIDPS group. Firstly, drug sensitivity data of cancer cell lines were obtained from the CTRP and PRISM reutilization data sets, and gene expression data of these cancer cell lines were obtained from the Cancer Cell Line Encyclopedia (CCLE) database. Secondly, the CTRP and PRISM data sets provided AUC values, with lower AUC values indicating increased sensitivity to the drugs. As a frontline chemotherapy drug for STAD, we further selected dasatinib to validate the scientific and reliable nature of this approach. Thirdly, differential analysis of drug response between the top 10% high AIDPS group and bottom 10% low AIDPS group was performed using the Wilcoxon rank‐sum test, with a threshold of log_2_FC >0.2 set to determine compounds with lower AUC values in the high AIDPS group. Fourthly, compounds with negative correlation coefficients between AUC values and AIDPS were further screened using Spearman correlation (threshold set at *R* < −0.4). Lastly, potential drugs applicable to high AIDPS group patients were determined by combining the compounds obtained from steps (3) and (4). The publicly available network tool CMap (https://clue.io/) was used to discover candidate compounds targeting AIDPS‐related pathways based on gene expression characteristics. Potential compounds were identified in STAD using CMap, based on differential expression analysis, to further validate the results obtained from the CTRP and PRISM databases.

### 2.9 Single‐cell analysis of STAD

Gene high‐throughput single‐cell sequencing data related to STAD was downloaded from the GEO database. By applying the filtering criteria: STAD, human, peripheral tissue and single cell RNA‐seq/scRNA‐seq, we obtained single‐cell sequencing data that met the filtering standards. In GSE150290, 10X scRNA‐seq was performed on cells from 24 STAD patients and 24 adjacent tissues. Quality control processing was carried out using Limma, Seurat, Dplyr and Magrittr packages in R, filtering out low‐abundance data. Subsequently, principal component analysis (PCA) was performed for dimensionality reduction and cluster analysis. Cell clustering annotation was conducted based on the expression levels of specific cell marker genes. DESeq2 and Wilcoxon tests were utilized to calculate differentially expressed genes between the Atherosclerosis group and Control group, with corresponding filtering conditions set. A heatmap of differentially expressed genes between different groups of cells was created using the Heatmap function. In further analysis, correction based on annotation results and differential analysis of AIDPS‐related genes were performed. Using cell–cell similarity matrices, the interaction of specific genes between cell populations was visualized, including cell pathway analysis, construction of cell interaction networks and co‐expression analysis. Through these analyses, we explored the impact of AIDPS in STAD, providing new insights and methods for the treatment and prevention of related diseases.

### 2.10 Cultivation of gastric adenocarcinoma cell lines and macrophages

The human gastric cancer cell lines SGC‐7901 and AGS, as well as macrophages (THP‐1), were purchased from the Shanghai Institute of Life Sciences. They were cultured in RPMI‐1640 medium containing 10% foetal bovine serum, 100 kU/L penicillin and 100 mg/L streptomycin at 37°C in a 5% CO_2_ incubator using standard cultivation procedures.

### 2.11 Cell transfection

An interference plasmid targeting the GHRL gene (sh‐GHRL) was constructed using shRNA and transfected into SGC‐7901 and AGS cells. The cells were divided into the following groups: SGC‐7901, AGS, SGC‐7901/sh‐GHRL, AGS/sh‐GHRL, SGC‐7901/sh‐NC and AGS/sh‐NC. The culture medium was replaced 6 h after transfection, and cells were harvested for real‐time PCR detection after 48 h and Western blot detection after 72 h. The sequence of sh‐GHRL is 5′‐CCCTTTGATGTTGGAATCAAG‐3′. The plasmid vector was PLKO.1‐puro (Youbia, China).

### 2.12 Co‐culture of macrophages with gastric adenocarcinoma cells

THP‐1 cells (5 × 10^5^ cells/mL) were seeded in a 6‐well plate and treated with 200 ng PMA to obtain undifferentiated M0 macrophages at a final concentration of 100 ng/mL. They were then co‐cultured with SGC‐7901, AGS, SGC‐7901/sh‐GHRL, AGS/sh‐GHRL, SGC‐7901/sh‐NC and AGS/sh‐NC, respectively. The cell groups were as follows: SGC‐7901/M0 macrophages, AGS/M0 macrophages, SGC‐7901/sh‐GHRL‐M0 macrophages, AGS/sh‐GHRL‐M0 macrophages, SGC‐7901/sh‐NC‐M0 macrophages and AGS/sh‐NC‐M0 macrophages.

### 2.13 Cell proliferation assay

The influence of GHRL on drug‐resistant gastric adenocarcinoma cells was evaluated using the CCK‐8 assay. A volume of 100 μL cell suspension was taken from AGS and SGC‐7901 cells treated with different conditions and seeded into a 96‐well plate. After 72 h, CCK‐8 reagent was added to the wells. Cell proliferation rates were calculated, and IC50 values were determined as the mean equivalent.

### 2.14 Macrophage phenotypic identification

Macrophage surface specific markers for M1/M2 phenotypes were detected using a flow cytometer. After induction, M‐type macrophages were collected by centrifugation at 1500 rpm for 5 min at 4°C, washed with PBS three times and resuspended in 100 μL of PBS. Antibodies were added according to the instructions of the flow cytometry antibody kit, and the cells were incubated on ice, protected from light, for 30 min. After washing and resuspending the cells in PBS, macrophage surface markers were detected using a flow cytometer. In addition, total RNA was extracted from each group of cells using the Trizol method as per the instructions. The cDNA was synthesized using a reverse transcription kit, and the expression of macrophage differentiation markers CD206 and CD163 genes was detected by PCR (primers are shown in Table 1). The PCR reaction conditions were as follows: 95°C for 30 s, 60°C for 30 s and 72°C for 30 s, for a total of 45 cycles. The relative expression levels of each gene's mRNA were calculated using the 2^−ΔΔCt^ method. The experiment was repeated three times, and the average value was taken. Different samples of cells from different experimental conditions were collected for protein extraction, and the expression levels of GHRL, M2 macrophage markers (such as CD206 and CD163) and other proteins between different groups were detected using Western blotting techniques. CD206 and CD163 antibodies were purchased from abcam.

**TABLE 1:** Primer sequences.

Gene	Forward primer (5′ to 3′)	Reverse primer (5′ to 3′)
GAPDH	AGAAGGCTGGGGCTCATTTG	AGGGGCCATCCACAGTCTTCT
SPINK4	TTCAGTGCACTGCCCTAAGG	CCTCGCAGCAAGTCCTTTTC
TTR	GTGGTCCGAGGATCTCCTTC	CAGGTTCCCCCACCTTTAAT
PCSK1N	TCTCCGCTCTTTTGAGGACA	GTCCCTTCTGCTCCTTTTCC
CHGA	GGATCCCTCCAGGAGATTTG	GAAACTGCGTGACAATCTCCA
GHRL	TTGGGATGTCCACACAGGTG	GAGGGGAGGAGAGAGGTAGTG
IL‐1β	CATCTTCAAATCTCACAGCAGCA	GTCGCTGGTTATCTCATCTGGAA
CD86	GAGACATCGCCCTTACTGAAA	TCTCACACCCCAGTATTTTGA
IL‐10	CTCCAGGTAACAGCCAGACT	ATGCTCCCCACGAAGACTGA
CD206	GAAAATGAATGCAGTGTGCTG	GGCATACTGCAGCCTGTTTC

### 2.15 Transwell invasion assay

An 8 μm pore size Transwell chamber was used, and 60 μL of Matrigel (1:8) was applied to the upper chamber. It was then incubated in a cell culture incubator for 30 min to allow gel formation. Organoid cells were trypsinized, resuspended in serum‐free DMEM medium and diluted to a concentration of 3 × 10^5^ cells/mL. A volume of 200 μL of the cell suspension was added to the upper chamber of the Transwell, while the lower chamber was seeded with the same number of tumour cells and filled with complete culture medium (containing 20% FBS) for 24 h of incubation. The chambers were then removed, fixed with methanol at room temperature for 15 min, stained with crystal violet for 20 min, washed with PBS three times and randomly selected five fields were photographed and counted under a microscope for statistical analysis. Each group was set up with three replicate wells, and the experiment was repeated three times.

### 2.16 Quantitative real‐time PCR (qRT‐PCR)

Total RNA was extracted from each group of cells using Trizol reagent. 1 mL of Trizol reagent was added to each well, and the content was transferred to a 1.5 mL EP tube for 10 min of lysis. 200 μL of chloroform was added to each tube, followed by centrifugation at 4°C and 12,000 rpm for 15 min. The upper aqueous phase was transferred to a new tube, and 400 μL of isopropanol was added. After multiple centrifugation steps, the supernatant was discarded, and the pellet was dissolved in 20 μL of DEPC water. The cDNA was synthesized at 25°C for 5 min, 50°C for 15 min, 85°C for 5 min, and then kept at 4°C for 10 min. The cDNA was then diluted 10‐fold, and real‐time fluorescence quantitative PCR amplification was performed using the qRT‐PCR reaction system. The primer sequences, with GAPDH as the reference gene, are shown in Table 1.

### 2.17 Western blotting

The samples from different groups were dissolved in RIPA buffer, and the protein concentration was determined using the BCA method. A total of 25 μg of protein from each sample was loaded onto a SDS‐PAGE gel for protein electrophoresis and transferred to a PVDF membrane. The membrane was blocked with 50 g/L skim milk for 1 h and then incubated with primary antibodies overnight at 4°C. After washing the membrane with TPBS, it was incubated with secondary antibodies at 37°C on a shaker for 2 h. Chemiluminescence, testing and photography were performed using the DNR Bio‐Imaging System, and Image J software was used for image analysis to calculate the relative expression levels of proteins in the organoid samples from each group. The GAPDH antibody used in the project was purchased from proteintech, and all other antibodies were purchased from abcam.

### 2.18 Statistical analysis

Each experiment was repeated three times. Data were analysed using GraphPad Prism 8.0.1 and SPSS 25.0 software. Results are expressed as mean ± standard deviation. Differences among multiple groups were compared using ANOVA analysis, while differences between two groups were compared using *t*‐tests. The test result with a *p*‐value less than 0.05 was considered statistically significant.

## 3 RESULTS

### 3.1 Molecular features in gastric adenocarcinoma identified by machine learning models

**FIGURE 1:**
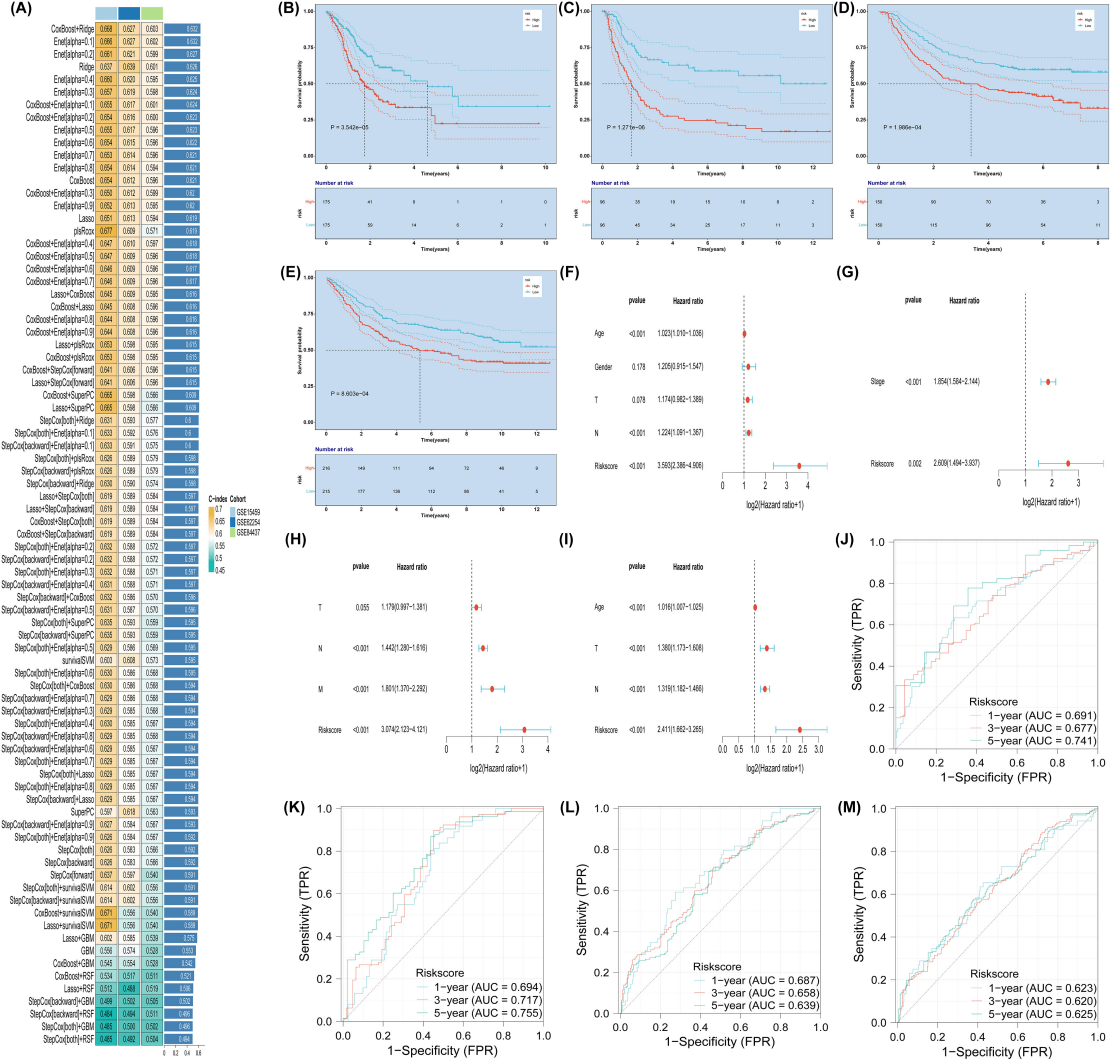
Molecular features identified by machine learning model in gastric adenocarcinoma (A). Consistent prognostic value of AIDPS (B). Survival prognostic curves in TCGA (C), GSE15459 (D), GSE62254 (E) and GSE84437 (F). Multivariable analysis of TCGA (G), GSE15459 (H), GSE62254 (I) and GSE84437 (J). ROC curves in TCGA (K), GSE15459 (L), GSE62254 (M).

Using the expression profiles of immune‐related genes, we finally identified 47 prognostic genes through univariate Cox analysis. These genes were then utilized in our machine learning‐based integrative procedure to develop an AIDPS. In the TCGA‐STAD data set, we employed the LOOCV framework to fit 101 prediction models and calculated the C‐index for each model across all validation data sets. Notably, the optimal model emerged as a combination of CoxBoost and Ridge, achieving the highest average C‐index of 0.632 and outperforming other models in all validation data sets. As shown in Figure 1A, CoxBoost+Ridge had the highest average C‐index (0.632) and was selected as the final model, resulting in 13 relevant genes, including PAEP, PROC, DKK1, GHRL, STC1, NOX4, NPR3, NRP1, CLCF1, ITGAV, SH3BP2, SEMA4G and TAP1.

### 3.2 Consistent prognostic value of AIDPS

To evaluate the prognostic performance of AIDPS, we divided STAD patients into high and low AIDPS groups based on the median score. In the STAD‐TCGA training set, the Kaplan–Meier curves for overall survival (OS) showed significantly shorter survival time in the high AIDPS group (*p* < 0.0001, Figure 1B). After removing batch effects, the same trend was observed in the three test sets, GSE15459, GSE62254 and GSE84437 (all *p* < 0.05, Figure 1C–E). Additionally, we included several important clinical features for multivariate Cox analysis, which demonstrated that AIDPS was an independent protective factor for OS in the STAD‐TCGA cohort (HR: 3.593, 95% CI: 2.386–4.906, Figure 1F). Similar results were found in the three test sets, GSE15459 (HR: 2.609, 95% CI: 1.494–3.937, Figure 1G), GSE62254 (HR: 3.074, 95% CI: 2.123–4.121, Figure 1H) and GSE84437 (HR: 2.411, 95% CI: 1.662–3.265, Figure 1I). To evaluate the discriminative ability of AIDPS, we plotted calibration curves and receiver operating characteristic (ROC) curves. The STAD‐TCGA training set demonstrated good predictive performance of AIDPS (Figure 1J), with AUC values of 0.691, 0.677 and 0.741 for 1‐year, 3‐year and 5‐year OS, respectively. Similarly excellent results were obtained in the three test sets, with AUC values for GSE15459 of 0.694, 0.717 and 0.755, for GSE62254 of 0.687, 0.658 and 0.639 and for GSE84437 of 0.623, 0.620 and 0.625 (Figure 1K–M). The AUC values exceeding 0.65 in multiple independent cohorts indicate that our AIDPS can reliably and consistently predict the prognosis of STAD patients.

### 3.3 Clinical features and potential biological mechanisms of AIDPS

**FIGURE 2:**
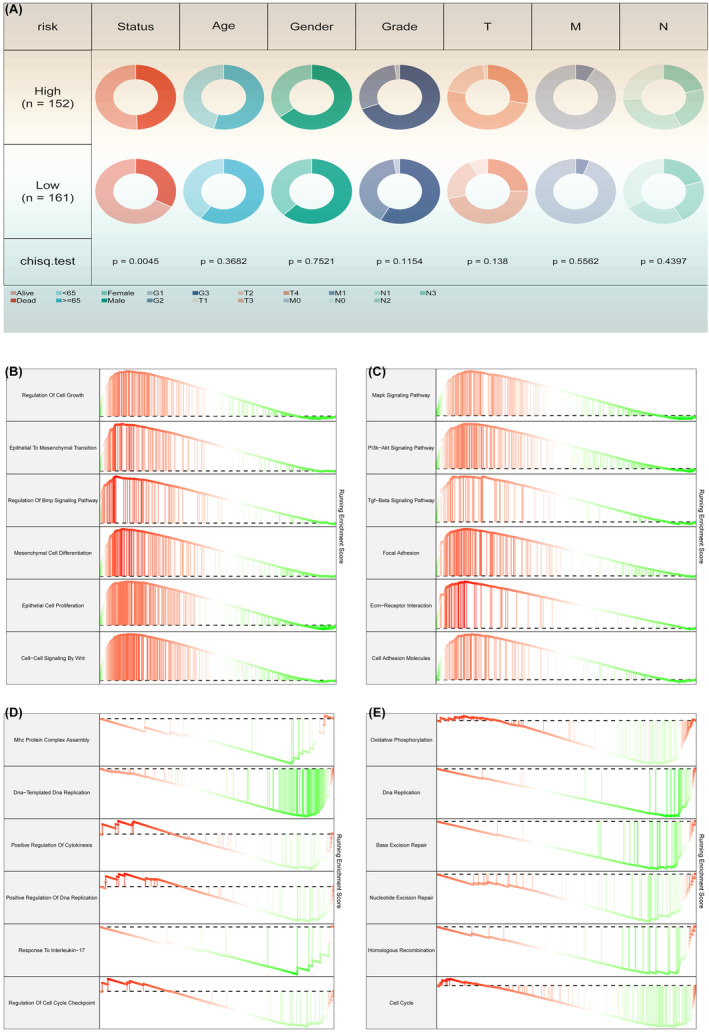
Clinical characteristics of AIDPS. (A) Analysis of clinical features. (B) Biological functional analysis of high AIDPS. (C) Signalling pathway analysis of high AIDPS. (D) Biological functional analysis of low AIDPS. (E) Signalling pathway analysis of low AIDPS.

We further compared several common clinical features between the high and low AIDPS groups and found no statistically significant differences in age, sex or tumour stage, but a strong correlation with patient survival status (*p* < 0.01, Figure 2A). However, patients in the high AIDPS group had worse survival status, which may contribute to their poor prognosis. Gene set enrichment analysis (GSEA) was performed to explore the potential functional pathways of AIDPS. As shown in Figure 2B,C, the high AIDPS group exhibited significantly enriched pathways in regulation of cell growth, epithelial to mesenchymal transition and regulation of Bmp signalling Pathway, including pathways such as Mapk signalling pathway, Pi3k‐Akt signalling pathway and Tgf‐Beta signalling pathway. On the contrary, the low AIDPS group was mainly associated with Mhc protein complex assembly, DNA‐templated DNA replication, positive regulation of cytokinesis and other immune‐related pathways, as well as immune‐related biological processes like oxidative phosphorylation, DNA replication and base excision repair. These findings partly explain the higher grade and worse prognosis in the low AIDPS group (Figure 2D,E).

### 3.4 Genomic alterations in AIDPS

**FIGURE 3:**
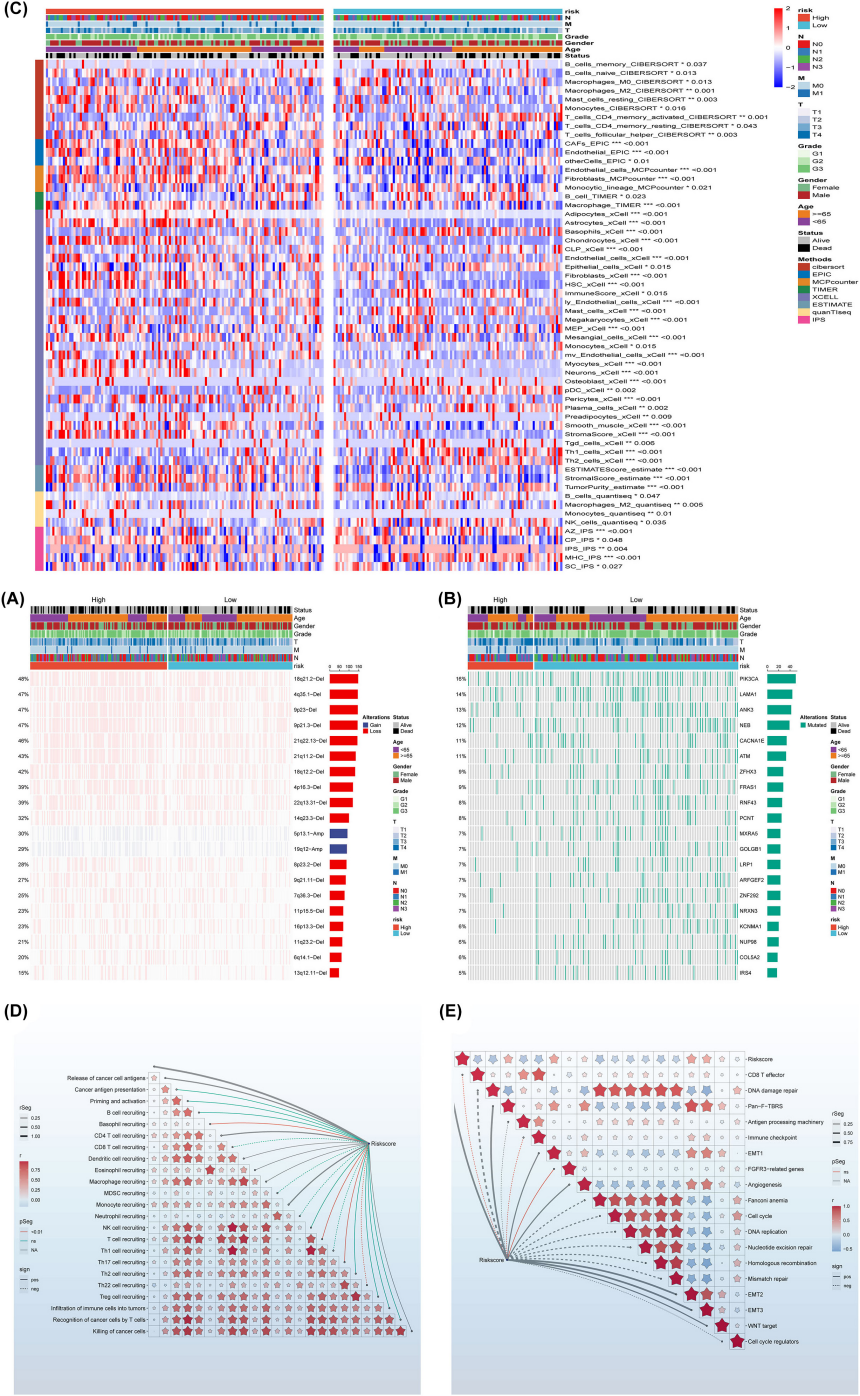
Genomic alterations, immune landscape and molecular expression in AIDPS. (A) Copy number variations. (B) Single nucleotide polymorphism differences. (C, D) Immune infiltrate analysis in AIDPS model. (E) Immune checkpoint analysis in AIDPS model.

To investigate the genomic heterogeneity between the high and low AIDPS groups, we conducted a comprehensive analysis of mutations and copy number variations (CNVs, Figure 3A). As shown in Figure 3A, the high AIDPS group exhibited a significantly higher tumour mutation burden (TMB). Analysis of single nucleotide polymorphisms revealed higher mutation frequencies of genes PIK3CA, LAMA1, ANK3, NEB and CACNA1E in the high AIDPS group (Figure 3B). Furthermore, we explored the CNV features of these two groups. Compared to the low AIDPS group, the high AIDPS group showed more frequent amplifications or deletions at the local and chromosomal arm levels, such as amplifications at 5p13.1 and 19q12 and deletions at 18q21.2, 4q35.1 and 9p23 (Figure 3A). Overall, the amplification of oncogenes and deletion of tumour suppressor genes in the high AIDPS group may contribute to their adverse prognosis.

### 3.5 Immune landscape and molecular expression in AIDPS

The GSEA analysis demonstrated significant enrichment of multiple immune‐related pathways in the high AIDPS group, prompting us to further investigate the immune landscape and expression of immune checkpoint molecules (ICMs) between the two groups. Based on single‐sample gene set enrichment analysis (ssGSEA), we found relatively higher levels of immune cell infiltration in the high AIDPS group, including B_cells_memory, B_cells_naive, M Macrophages_M0 and Macrophages_M2 (all *p* < 0.05, Figure 3C,D). Additionally, the relative expressions of 19 ICMs were significantly increased in the low AIDPS group, such as FGFR3‐related genes, EMT1, immune checkpoint, antigen processing machinery, Pan‐F‐TBRS, DNA damage repair and CD8 T effector (Figure 3E). Collectively, these consistent findings suggest that STAD patients with high AIDPS expression are more likely to respond to immune therapy.

### 3.6 Predictive value and potential therapeutic drugs of immune therapy in high AIDPS group

**FIGURE 4:**
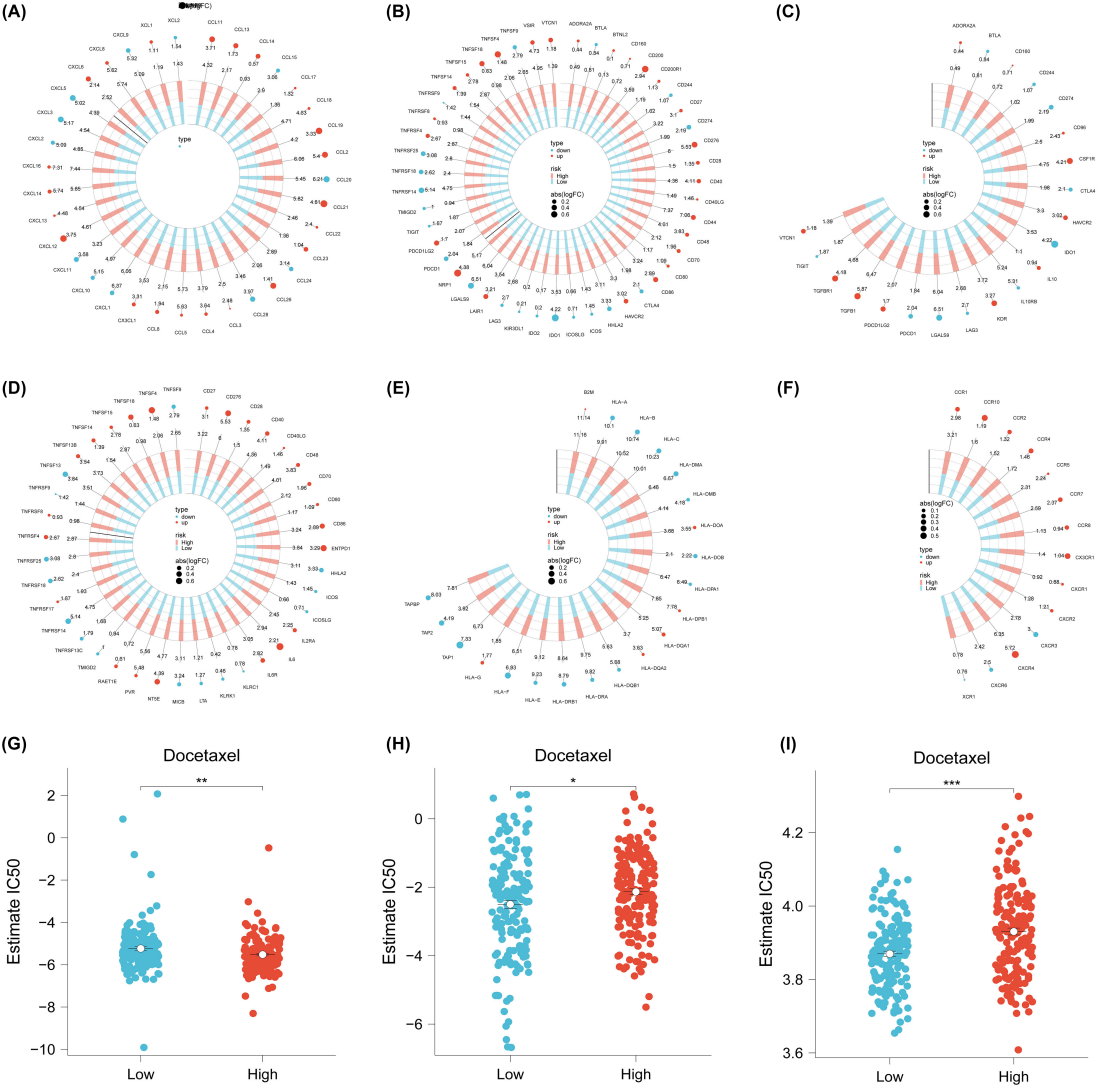
Predictive value of immune therapy and potential therapeutic drugs in the high AIDPS group. (A–F) Immune therapy sensitivity in the AIDPS model. (G–I) Potential drug screening in the AIDPS model (*p < 0.05; **p < 0.01; ***p < 0.001).

Considering that patients in the high AIDPS group have a higher frequency of genomic variations and tumour mutation burden (TMB), as well as a relatively activated tumour microenvironment (TME) and increased immune checkpoint molecule (ICM) expression, we speculate that STAD patients in the high AIDPS group are more likely to develop resistance to immune therapy. According to the results of tumour immune checkpoint inhibition assessment, the immune checkpoint inhibition scores in the low AIDPS group are significantly lower, indicating a higher response rate to immune therapy (Figure 4A–F). Overall, these results suggest that the low AIDPS group is more likely to benefit from immune therapy. As shown in Figure 4G–I, we used sensitivity data from the Cancer Therapeutics Response Portal (CTRP) data set (including 481 compounds from 835 cancer cell lines [CCL]) and the PRISM data set (including 1448 compounds from 482 CCL) to develop potential treatment options for STAD patients in the high AIDPS group. As a result, three CTRP‐derived drugs (docetaxel, gemcitabine and sorafenib) were generated.

### 3.7 Differential expression of AIDPS in STAD macrophages

**FIGURE 5:**
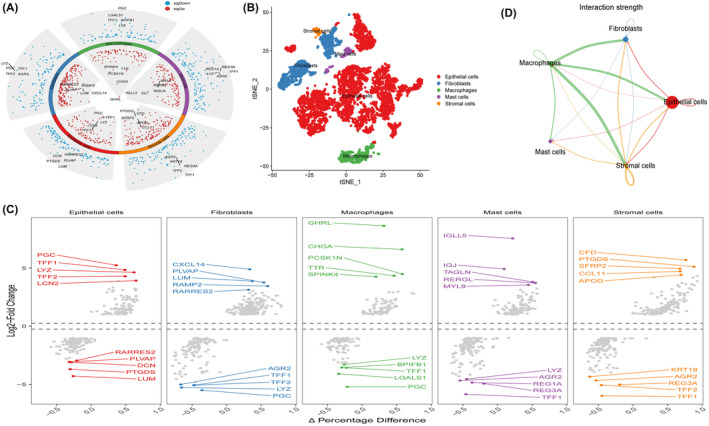
Differential expression of AIDPS in STAD epithelial cells. (A) TSNE distribution of cells after cell annotation. (B) Expression pattern of marker genes in five cell clusters. (C) Distribution of AIDPS‐related genes in STAD single‐cell clusters. (D) Cell communication analysis.

Using known marker genes, STAD cell clusters were annotated and subjected to t‐SNE dimensionality reduction clustering analysis. The annotated cells were divided into five distinct clusters: epithelial cells, fibroblasts, macrophages, mast cells and stromal cells (Figure 5A). The average expression levels of the AIDPS genes obtained from the above analysis were calculated as marker genes for each cluster (Figure 5B). The distribution of the marker genes in the five cell clusters was further analysed using the Seurat package (Figure 5C). We also analysed the communication patterns and strengths between different cells in the experimental and control groups. We found that there were differences in the interactions between cells from different groups (Figure 5D).

### 3.8 Upregulation of AIDPS‐related genes in gastric adenocarcinoma cell lines

**FIGURE 6:**
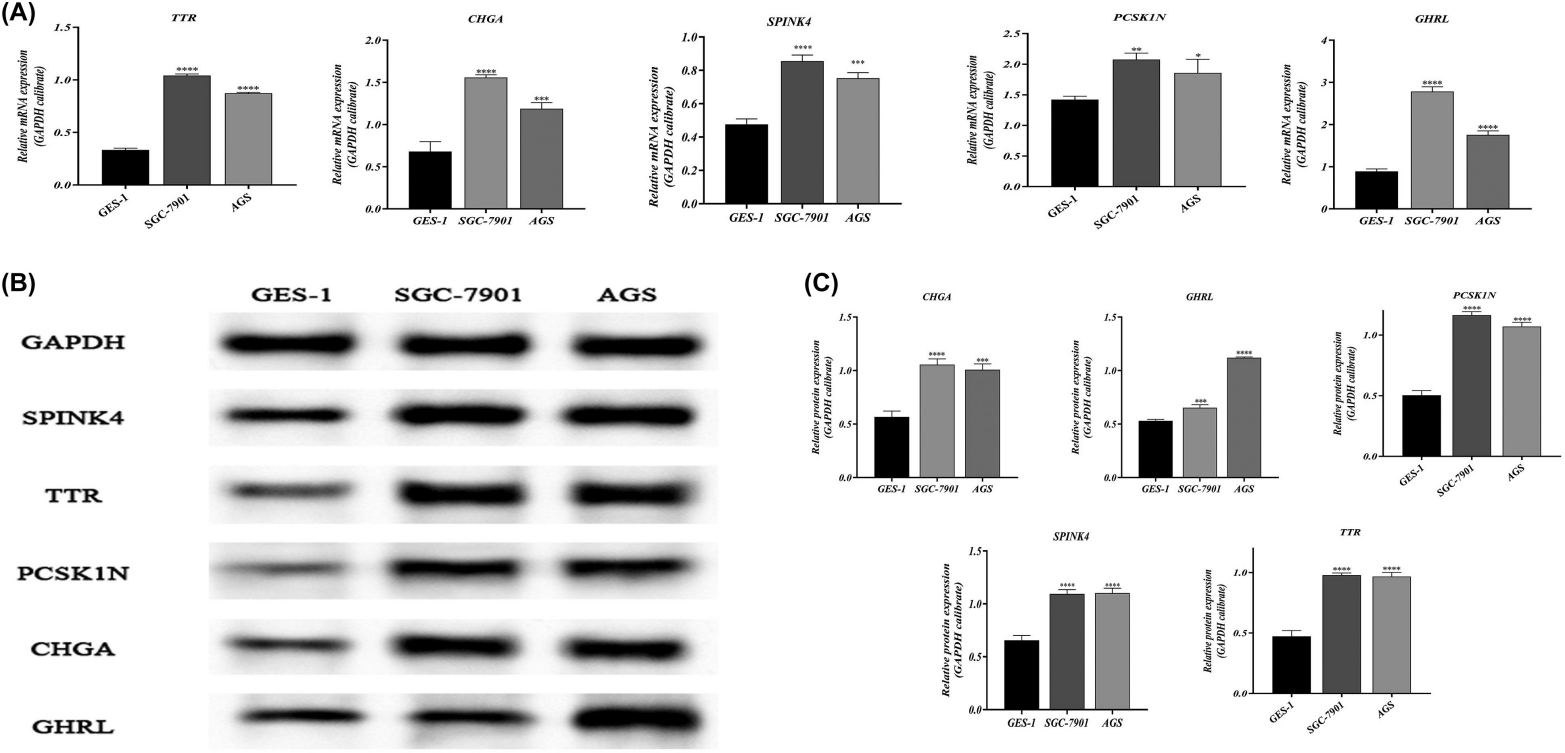
Upregulation of AIDPS‐related genes in gastric adenocarcinoma cell lines. (A) qPCR analysis of AIDPS‐related gene expression. (B) Western blot bands of AIDPS‐related protein expression. (C) Quantification of AIDPS‐related protein expression (*p < 0.05; **p < 0.01; ***p < 0.001; ****p < 0.0001; vs. GES‐1 cells).

RT‐qPCR and Western blotting were performed to analyse the expression of AIDPS‐associated genes in human gastric cancer cell lines SGC‐7901 and AGS. The results indicated that the mRNA expression levels of SPINK4, TTR, PCSK1N, CHGA and GHRL were higher in SGC‐7901 and AGS cell lines compared to normal cell lines (*p* < 0.05, Figure 6A). Western blotting also showed high expression of SPINK4, TTR, PCSK1N, CHGA and GHRL in gastric adenocarcinoma cell lines, with GHRL showing the most significant increase (*p* < 0.05, Figure 6B,C).

### 3.9 sh‐GHRL suppresses proliferative activity of gastric adenocarcinoma cell lines

**FIGURE 7:**
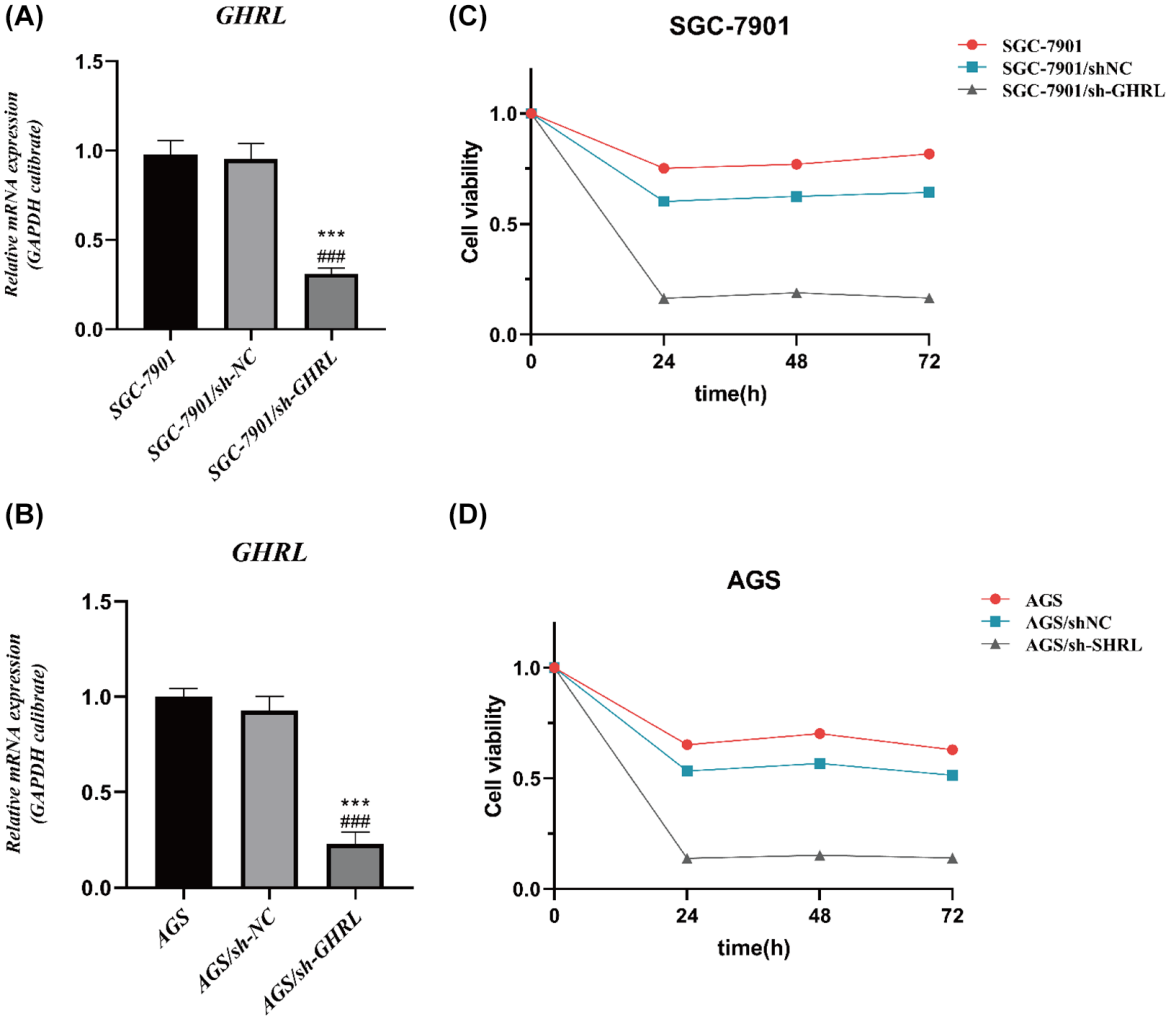
Inhibition of cell proliferation activity in gastric adenocarcinoma cell lines by sh‐GHRL (CCK‐8 results). (A, B) Detection of mRNA expression of GHRL in different groups by qRT‐PCR. (C) Inhibition of cell proliferation activity in SGC‐7901 gastric adenocarcinoma cell line by sh‐GHRL. (D) Inhibition of cell proliferation activity in AGS gastric adenocarcinoma cell line by sh‐GHRL (*p < 0.05, **p < 0.01, ***p < 0.001, ****p < 0.0001, vs. SGC‐7901 or AGS group; ^###^ *p* < 0.001, ^####^ *p* < 0.0001, vs. SGC‐7901/sh‐NC or AGS/sh‐NC group).

**FIGURE 8:**
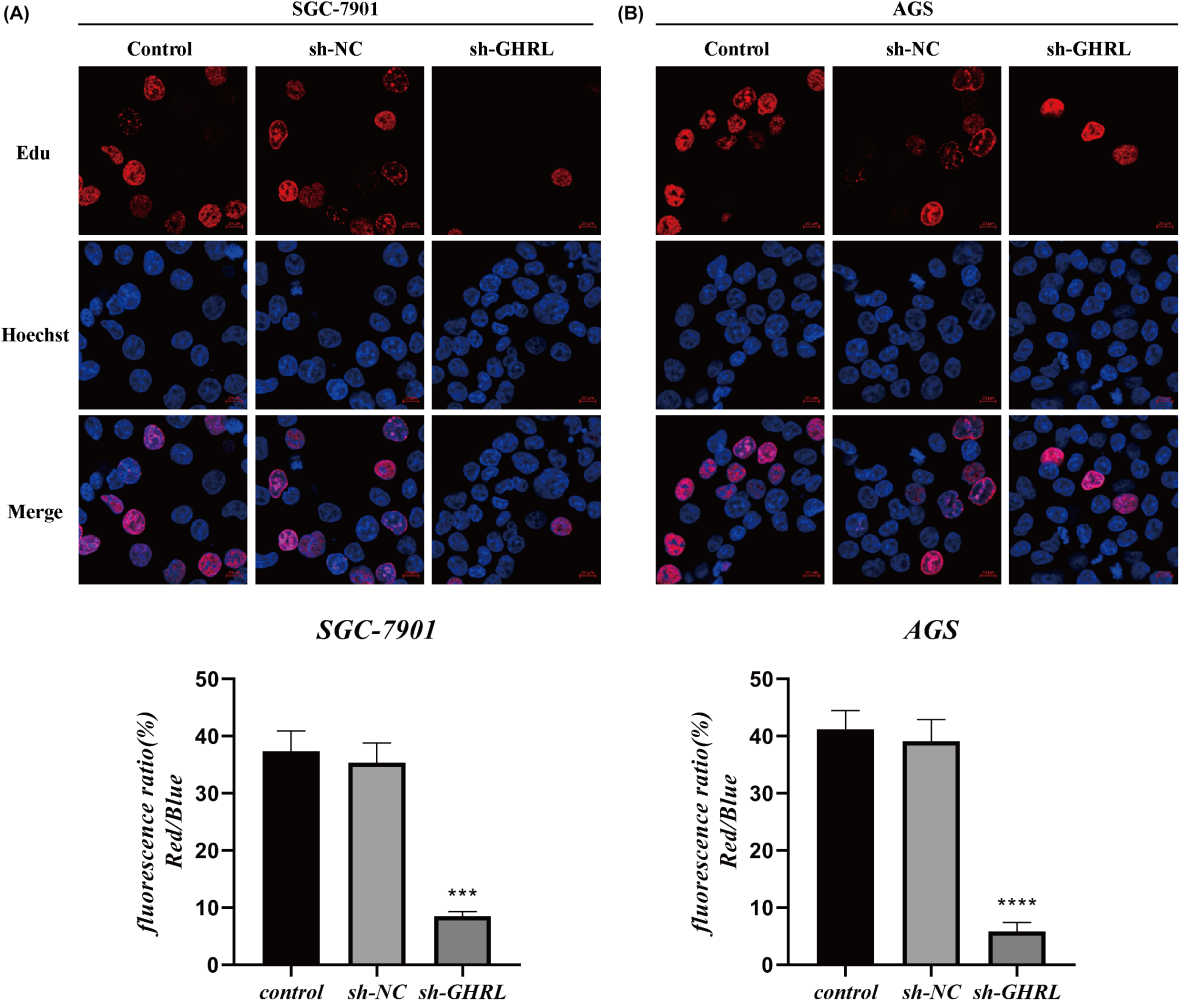
Inhibition of cell viability in gastric adenocarcinoma cell lines by sh‐GHRL (Edu results). (A) Inhibition of cell viability in SGC‐7901 gastric adenocarcinoma cell line by sh‐GHRL. (B) Inhibition of cell viability in AGS gastric adenocarcinoma cell line by sh‐GHRL (*p < 0.05; **p < 0.01; ***p < 0.001; ****p < 0.0001).

To investigate whether inhibiting GHRL expression affects the proliferative activity of gastric adenocarcinoma cell lines, firstly, we constructed stabilized cell lines of AGS and SGC‐7901 that silenced GHRL and detected the expression of GHRL by qRT‐PCR (Figure 7A,B). CCK8 assay was performed to assess the activity of gastric adenocarcinoma cell lines upon GHRL knockdown. As shown in Figure 7C,D, it can be observed that inhibiting GHRL expression decreased the activity of SGC‐7901 and AGS gastric adenocarcinoma cell lines. Additionally, the results of the Edu assay were consistent with the CCK8 assay results, as shown in Figure 8. These findings suggest that inhibiting GHRL expression suppresses the proliferation of gastric adenocarcinoma cell lines.

### 3.10 Inhibition of GHRL suppresses M2 polarization of gastric adenocarcinoma macrophages

**FIGURE 9:**
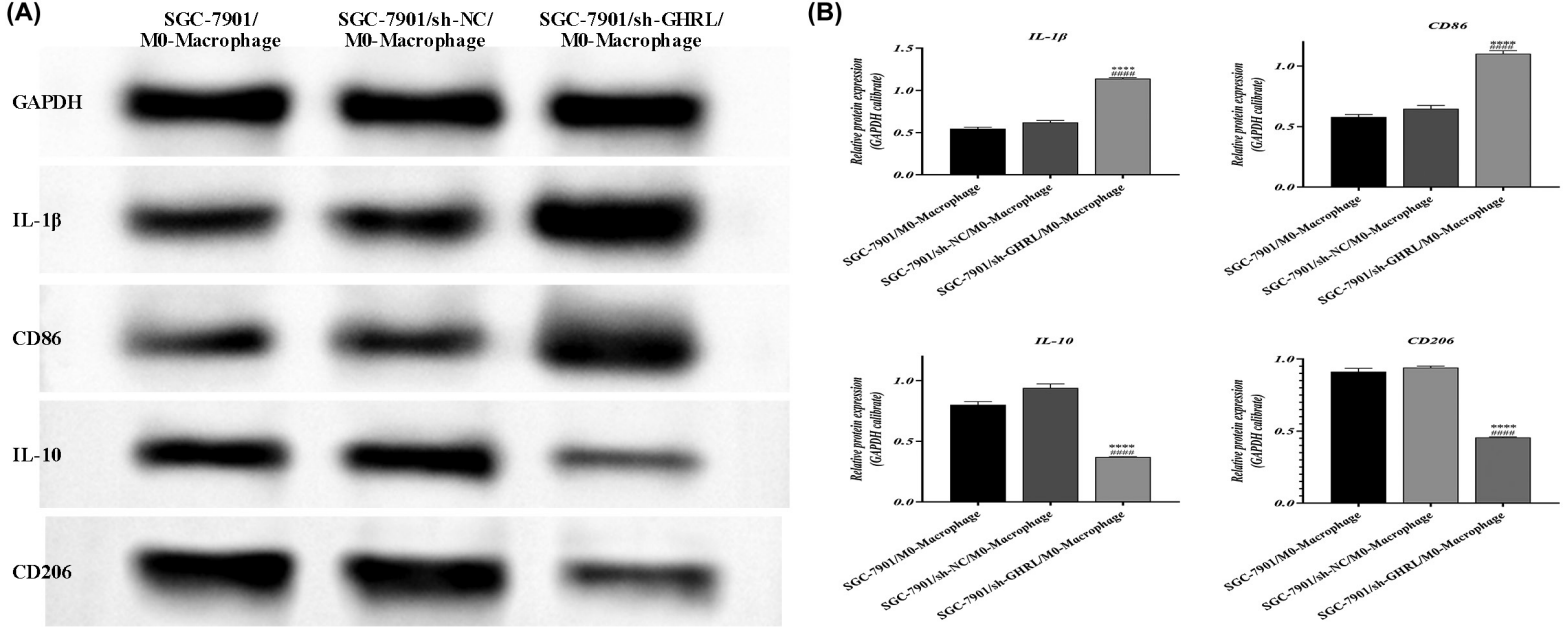
Inhibition of GHRL suppresses M2 polarization of gastric adenocarcinoma SGC‐7901 cells‐macrophages. (A) Western blot bands of M1/M2 polarization markers in different groups of cells. (B) Quantification of protein expression (*p < 0.05, **p < 0.01, ***p < 0.001, ****p < 0.0001, vs. SGC‐7901/M0‐Macrophage group; ^####^ *p* < 0.0001, vs. SGC‐7901/sh‐NC/M0‐Macrophage group).

**FIGURE 10:**
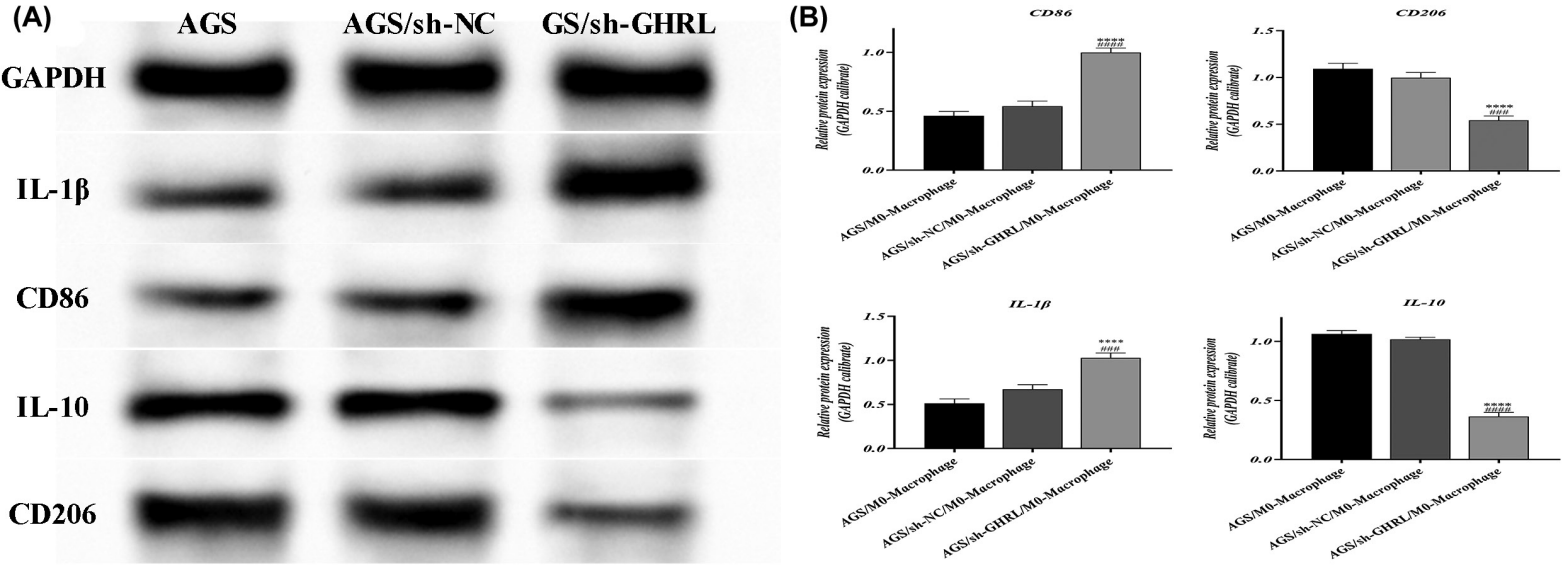
Inhibition of GHRL suppresses M2 polarization of gastric adenocarcinoma AGS cells‐macrophages. (A) Western blot bands of M1/M2 polarization markers in different groups of cells. (B) Quantification of protein expression (*p < 0.05, **p < 0.01, ***p < 0.001, ****p < 0.0001, vs. AGS/M0‐Macrophage group; ^###^ *p* < 0.001, ^####^ *p* < 0.0001, vs. AGS/sh‐NC/M0‐Macrophage group).

Human monocytic cell line THP‐1 was treated with PMA to differentiate into macrophages. Furthermore, the differentiated macrophages were assessed for marker changes using Western blot. Compared to the sh‐NC group, the expression of M2 macrophage markers IL‐10 and CD206 decreased, while the expression of M1 markers IL‐1β and CD86 increased in the sh‐GHRL group (*p* < 0.01, Figures 9 and 10). These results indicate that downregulation of GHRL inhibits M2 polarization of tumour‐associated macrophages.

### 3.11 sh‐GHRL suppresses cell invasion of gastric adenocarcinoma cell lines

**FIGURE 11:**
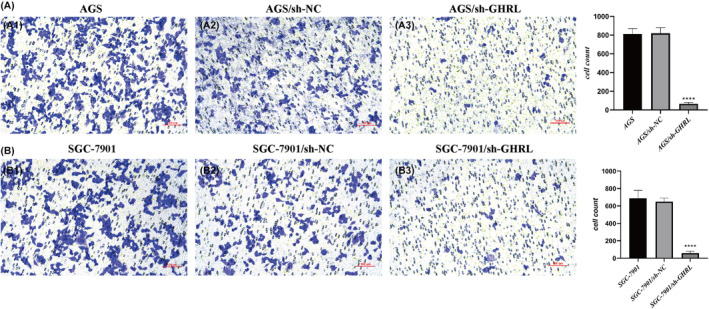
Inhibition of cell invasion in gastric adenocarcinoma cell lines by sh‐GHRL. (A) AGS cells. (B) SGC‐7901 cells (****p < 0.0001).

The ability of cell invasion upon GHRL interference was validated using the transwell assay, as shown in Figure 11. It was evident that inhibiting GHRL significantly suppressed the invasive capacity of gastric adenocarcinoma cell lines. These findings indicate that interfering with GHRL inhibits the invasiveness of gastric adenocarcinoma cell lines.

## 4 DISCUSSION

Significant progress has been made in the field of gastric cancer research. However, there are still challenges and limitations in treatment that need to be addressed. The complexity of gastric cancer restricts the efficacy of treatment strategies, with traditional methods such as surgical resection and chemotherapy showing good results for early‐stage patients but often failing to achieve desired outcomes for advanced‐stage patients.^17^ Additionally, the development of chemoresistance poses a challenge for clinical treatment.^18^ Therefore, the development of new therapeutic approaches is crucial to improve survival rates and quality of life for gastric cancer patients. In recent years, immunotherapy has emerged as a promising treatment modality, bringing new hope for gastric cancer management. By activating the patient's immune system, immunotherapy can target tumour cells and elicit antitumour effects.^19^, ^20^ However, there is significant interindividual variability in the response and tolerability of gastric cancer patients to immunotherapy, with some benefiting significantly while others fail to achieve the desired therapeutic effects.^21^, ^22^ Consequently, understanding the immune molecular characteristics and immune escape mechanisms of gastric cancer patients is of paramount importance for individualized design and prediction of immunotherapy efficacy.

In this study, a gastric adenocarcinoma machine learning model (AIDPS model) was constructed using cross‐validation and machine learning methods. It was found that CoxBoost+Ridge, with the highest average C‐index (0.632), was selected as the final model. Thirteen relevant genes were identified, including: PAEP, PROC, DKK1, GHRL, STC1, NOX4, NPR3, NRP1, CLCF1, ITGAV, SH3BP2, SEMA4G and TAP1. The AIDPS model has significant clinical implications for STAD, as patients with high AIDPS have a poorer prognosis, higher genomic alterations, more intense immune cell infiltration and increased tolerance to immunotherapy. DKK1 (Dickkopf‐1) is a molecule that is overexpressed in gastric adenocarcinoma and affects cell proliferation and metastasis by inhibiting the activation of the Wnt signalling pathway.^23^, ^24^ GHRL (Ghrelin) is a hormone that is highly expressed in gastric adenocarcinoma and is involved in tumour development by binding to its receptor, GHSR. It may promote tumour progression by enhancing gastric adenocarcinoma cell proliferation, inhibiting apoptosis and promoting angiogenesis.^25^, ^26^ STC1 (Stanniocalcin 1) is a protein that is highly expressed in gastric adenocarcinoma. It may influence the malignant transformation of tumours by promoting the growth, invasion and metastasis of gastric adenocarcinoma cells.^27^, ^28^ NOX4 (NADPH oxidase 4) is an enzyme that generates reactive oxygen species and is overexpressed in gastric adenocarcinoma. It may promote the development of gastric adenocarcinoma by increasing oxidative stress and reactive oxygen species production.^29^, ^30^ NRP1 (Neuropilin‐1) is a receptor that is overexpressed in gastric adenocarcinoma. It may promote tumour metastasis and development by regulating the movement and angiogenesis of gastric adenocarcinoma cells.^31^, ^32^ ITGAV (Integrin Alpha V) is a protein that is overexpressed in gastric adenocarcinoma. It may promote tumour progression by increasing the invasive ability and transmembrane signalling of gastric adenocarcinoma cells.^33^ In summary, molecules such as DKK1, GHRL, STC1, NRP1 and ITGAV play important roles in the development and prognosis of gastric adenocarcinoma.

In addition, this study found differential expression of AIDPS‐related genes in STAD macrophages through single‐cell analysis. M2 macrophages are an immune‐suppressive subpopulation of macrophages, and their polarization state is promoted in the tumour microenvironment, thereby affecting the development and progression of gastric adenocarcinoma.^34^ The polarization state of M2 macrophages promotes tumour angiogenesis and progression in the tumour microenvironment by releasing cytokines, such as pro‐angiogenic factors and growth factors.^35^ M2 macrophages can also produce anti‐inflammatory and apoptosis‐inhibiting factors, suppressing the activity of immune cells and weakening the presentation of tumour antigens and the immune system's killing effect on tumours.^36^ M2 macrophages can secrete matrix metalloproteinases and detachment‐related factors that promote tumour cell invasion and metastasis by disrupting the basement membrane, as well as enhancing tumour cell invasion and metastatic abilities through interactions with gastric adenocarcinoma cells, releasing cytokines and chemokines.^37^, ^38^ Anti‐inflammatory cytokines and immune‐suppressive molecules produced during the polarization of M2 macrophages, such as anti‐inflammatory cytokine IL‐10 and anti‐inflammatory cytokine TGF‐β, suppress the activity of immune cells such as T cells and natural killer cells, weakening their attack against tumour cells and promoting immune escape of the tumour. The polarization of M2 macrophages plays a significant role in the pathogenesis of gastric adenocarcinoma, and therapeutic strategies targeting M2 macrophage polarization may help improve the treatment outcomes of gastric adenocarcinoma.

Our study using a gastric adenocarcinoma cell line model revealed a high expression of GHRL in gastric adenocarcinoma cells. However, we observed that interference with GHRL significantly inhibited M2 polarization of macrophages, suppressed their invasion ability, and slowed down the progression of gastric adenocarcinoma. The GHRL gene may influence the pathogenesis of gastric adenocarcinoma by modulating M2 macrophage polarization and regulating the activity of macrophages and changes in the tumour microenvironment. GHRL gene may also participate in regulating the development and progression of gastric adenocarcinoma by influencing epithelial–mesenchymal transition and epigenetic regulation, and affecting the epithelial and mesenchymal characteristics of tumour cells. Through the polarization of M2 macrophages the anti‐inflammatory cytokines and immune‐suppressive molecules produced by polarized M2 macrophages, such as TGF‐β and IL‐10, can inhibit the activity of immune cells and reduce their recognition and killing effect on tumour cells, leading to immune escape of the tumour. In summary, the GHRL gene may affect the pathogenesis of gastric adenocarcinoma by regulating M2 macrophage polarization. It plays an important regulatory role in the development and progression of gastric adenocarcinoma by modulating the tumour microenvironment, promoting tumour invasion and metastasis and influencing immune escape. Future studies will require a deeper understanding of the interaction mechanisms between the AIDPS model and gastric adenocarcinoma to develop new treatment strategies for gastric adenocarcinoma.

In conclusion, the AIDPS model holds important clinical significance for STAD. Patients with high AIDPS scores have poor prognosis, higher genomic alterations, more intense immune cell infiltration and increased tolerance to immunotherapy. Inhibiting the expression of GHRL in AIDPS significantly suppresses the activity of gastric adenocarcinoma cell lines and inhibits the transition of macrophages to the M2 polarization state, thereby slowing the progression of gastric adenocarcinoma.

## AUTHOR CONTRIBUTIONS

**Wenli Chen:** Data curation (equal). **Xiaohui Liu:** Visualization (equal). **Houhong Wang:** Writing – review and editing (equal). **Jingyou Dai**: Visualization (equal). **Changquan Li**: Data curation (equal). **Dandan Jiang:** Writing – review and editing (equal).

## FUNDING INFORMATION

Bozhou Municipal Bureau of Science and Technology (BZZC2023046; BZZC2023045). Bozhou Municipal Health Commission Subjects (BZWJ2023A004). Bengbu Medical University Research Program (2023BYZD202). Anhui Medical University Doctoral Research Fund (BY2022015). Key Projects of Anhui Provinical Department of Education (2023AH050658).

## CONFLICT OF INTEREST STATEMENT

The authors declare no conflicts of interest related to this study.

## Data Availability

The data that support the findings of this study are available on request from the corresponding author. The data are not publicly available due to privacy or ethical restrictions.
